# Preparation and Adsorption Properties of Graphene-Modified, Pitch-Based Carbon Foam Composites

**DOI:** 10.3390/polym14204455

**Published:** 2022-10-21

**Authors:** Hao Li, Tiehu Li, Weibin Deng, Siyuan Kong

**Affiliations:** School of Materials Science and Engineering, Northwestern Polytechnical University, Xi’an 710072, China

**Keywords:** graphene, pitch-based carbon foam, composites, preparation, adsorption properties

## Abstract

In view of the good adsorption properties of graphene and carbon foam, they were combined to achieve the optimal matching of microstructures. Taking mesophase pitch as a raw material, pitch-based carbon foam was prepared by the self-foaming method. Graphene gel was prepared as the second phase to composite with the carbon foam matrix; graphene-modified, pitch-based carbon foam composites were finally obtained. Graphene gel was dispersed in the rich pore structure of carbon foam to improve its agglomeration and the porosity, and the active sites of the composite were further increased; the adsorption properties and mechanical properties of the composites were also significantly improved. The microstructure and morphology of the composites were studied by SEM, XRD and Raman spectroscopy; the compressive property and porosity were also tested. Methylene blue (MB) solution was used to simulate a dye solution for the adsorption test, and the influence of the composite properties and MB solution on the adsorption property was studied. Results showed that the compressive strength of the composite was 13.5 MPa, increased by 53.41%, and the porosity was 58.14%, increased by 24.15%, when compared to raw carbon foam. When the mass of the adsorbent was 150 mg, the initial concentration of the MB solution was 5 mg/L, and the pH value of the MB solution was 11; the graphene-modified carbon foam composites showed the best adsorption effect, with an adsorption rate of 96.3% and an adsorption capacity of 144.45 mg/g. Compared with the raw carbon foam, the adsorption rate and adsorption capacity of the composites were increased by 158.18% and 93.50%, respectively.

## 1. Introduction

With the continuous maturity of industrialization and economic development, the pollution of air, water and soil waste has become increasingly serious, especially water pollution, which has caused great impact on the human living environment and has become a key problem to be solved [1]. According to statistics, nearly 10,000 different synthetic dyes are widely used in textile processing; about 30% of dyes are discharged into the water source, affecting the water ecosystem seriously [2,3,4]. Therefore, it is necessary to adsorb and remove dye pollutants in order to separate and degrade them before they reach the water source [5,6]. Common dye wastewater treatment methods mainly include adsorption, oxidation, biodegradation, etc. [7,8,9,10,11,12,13,14]. The adsorption method is of great significance because it can selectively adsorb some compounds or dyes [15,16]. Carbon foam has become one of the indispensable materials in the adsorption field due to its porosity, simple preparation process, small density, large specific surface area and wide availability of raw materials (precursors) [17,18,19]. In recent years, researchers have increasingly studied the adsorption properties of carbon foam matrix composites. Udayakumar et al. [20] obtained carbon foam by heat treatment with polyurethane as the framework and activated carbon as the second phase. The carbon foam had both the porous network structure of polyurethane and the adsorption function of activated carbon, which is an ideal filter material. Chen et al. [21] took modified cyanate ester resin as the precursor, added nano-magnesium oxide as the pore nucleating agent and, finally, prepared multilayered carbon foam material through a series of heat treatment processes. The porosity of this material can reach 95.5% at most, and it has good selective adsorption to oil. Zbair et al. [22] prepared a new type of structural carbon foam for removing bisphenol A (BPA) from water. Results showed that the removal rate of BPA with a concentration of 60 mg/L by this carbon foam was 93%, and the regeneration experiment showed that the carbon foam is an economic and effective adsorbent. Galán et al. [23] developed a new type of mesoporous carbon (MCSG60). The experiment showed that the material has a high specific surface area and pore volume and explored the adsorption performance of the material for naphthol blue black (NBB), reactive black 5 (RB5) and Remazol brilliant blue R (RBBR). The experimental structure showed that the material has excellent adsorption performance for NBB, RB5 and RBBR reactive dyes. Nowadays, discovering how to further optimize the microstructure of carbon foam material and improve its adsorption performance is the focus of current research.

Graphene is an excellent adsorption material due to its large specific surface area and unique, two-dimensional lamellar structure [24,25]. The effects of graphene adsorbents on pollutants can be divided into hydrophilic adsorption and hydrophobic adsorption. Hydrophilic adsorption mainly refers to the epoxy functional group of graphene oxide, which is easier to combine with the hydrophilic group to achieve adsorption and which can effectively adsorb and remove a variety of toxic and harmful pollutants in water. Graphene is mainly a “hydrophobic group”, which is more inclined to combine with hydrophobic substances and can effectively adsorb pollutants such as oil [26,27]. Electrostatic adsorption, van der Waals force, dispersion and charge transfers are the main ways for graphene to adsorb pollutant molecules [28]. Zhou et al. [29] prepared a terpyridine/graphene composite successfully; test results showed that the specific surface area of the composite is 440 m^2^/g, and the carbon dioxide adsorption capacity is 12 mol/kg. Feng et al. [30] prepared highly reusable graphene oxide/oxidized cellulose nanofiber/polyvinyl alcohol aerogels with excellent properties, which showed a high adsorption capacity for various types of organic solvents. Jang et al. [31] prepared graphene composite membranes, which significantly increase the removal ability of the methylene blue and methyl red dyes in the aqueous system. However, the adsorption materials prepared from pure graphene have relatively weak structural stability and are easily damaged during use. At the same time, its interior is also prone to secondary agglomeration. Thus, discovering how to maintain the structural integrity of graphene adsorption materials and avoid their internal agglomeration is the focus of current researchers.

Based on the broad application potential of graphene and carbon foam in the adsorption field, and taking full advantage of their adsorption properties, graphene-modified, pitch-based carbon foam composites were synthesized in this study. Using mesophase pitch as a raw material, the self-foaming method was adopted to prepare carbon foam, and graphene gel was prepared as the second phase to composite with the carbon foam matrix. On the basis of the good adsorption performance of carbon foam, by increasing the material porosity and the number of surface active sites, graphene gel was dispersed in the rich pore structure of carbon foam to improve its agglomeration, which significantly improved the adsorption performance and mechanical properties of the composite and formed an optimized adsorption system. Test results showed that, when the adsorbent mass was 150 mg, the initial methylene blue (MB) concentration was 5 mg/L, and the pH value of MB solution was 11; the prepared graphene-modified, pitch-based carbon foam composite showed an adsorption rate of 96.32% and an adsorption capacity of 144.45 mg/g for MB, which reveals a wide application prospect in the field of water pollution treatment.

## 2. Materials and Methods

### 2.1. Materials

Coal tar pitch as carbon foam precursor was purchased from Koppers Carbon & Chemical Co., Ltd. (Pizhou, China); flake graphite as the raw material of graphene was purchased from Nanjing XFNANO Materials Tech Co., Ltd. (Nanjing, China).

### 2.2. Preparation of Graphene Gel

Graphene oxide (GO) was obtained by modified Hummers’ methods. First, we took 1 g of flake graphite and 1 g of NaNO_3_, added 50 mL of concentrated sulfuric acid into a beaker containing a mixture of flake graphite and NaNO_3_ and stirred it magnetically in a water bath for 30 min. Then, we added 6 g of KMnO_4_ slowly into the beaker, put the solution in water bath at 35 °C for 1 h, added 80 mL of distilled water slowly and heated and stirred continually at 95 °C for 0.5 h. Then, we added 80 mL of distilled water when the water bath decreased to room temperature and added 6 mL of H_2_O_2_ after stirring for 15 min; the solution turned bright yellow, and we adjusted the pH value of the solution to 7. Then, graphene oxide solution was obtained. We added L-cysteine to the graphene oxide solution and treated it ultrasonically for 2 h; the mass ratio of graphene to L-cysteine varied from 0.5 to 2.0 and then we put the solution in a water bath at 95 °C for 1 h to obtain graphene gel.

### 2.3. Preparation of Carbon Foam

Carbon foam was prepared from coal tar pitch by self-foaming method. First, coal tar pitch was thermally condensed to prepare mesophase pitch. An 8 g amount of ball-milled mesophase pitch was placed in a high-pressure reactor vessel, filled with nitrogen to 1.5 MPa, heated to 300 °C for 1 h and then the temperature was raised to 480 °C for 4 h. After that, the sample was taken out for carbonization. We placed the sample in the tubular furnace, with argon as the protective gas with ventilation rate 0.2 L/min, heated it to 600 °C for 1 h and then raised the temperature to 900 °C for 2 h. After this program was finished, the tubular furnace was cooled naturally to room temperature to obtain carbon foam material.

### 2.4. Preparation of Graphene-Modified Carbon Foam Composites

Carbon foam was cut into 50 mg, 75 mg, 100 mg, 125 mg and 150 mg samples; after ultrasonic cleaning, these samples were immersed into graphene gel for 48 h and then put in a vacuum drying oven at 70 °C for 48 h to obtain graphene-modified carbon foam composites.

### 2.5. Characterization of Graphene-Modified Carbon Foam Composites

Scanning electron microscopy (SEM, FEI Helios, Hillsboro, OR, USA) was used to analyze the morphology and microstructure of the composite. The crystal structure of the samples was characterized by X-ray diffractometer with Cu target (XRD, Bruker D8, Billerica, MA, USA). A Raman spectrometer (Raman, Alpha300R, Ulm, Germany) was used to characterize the microstructure and defects of samples.

The universal testing machine (GNT100, NCS Testing Technology Co., Ltd., Beijing, China) was used to test the mechanical properties of graphene-modified carbon foam composites. First, the composite materials were cut into small samples with the dimension 10 mm × 10 mm × 10 mm; these samples were compressed between two stainless platens with a loading rate of 0.5 mm/min, which is widely adopted in the compression strength testing of carbon foam [32,33], and then the stress–strain curve of the samples was obtained, as well as the failure load P and the compression section area A. Finally, compression strength σ of these samples was calculated according to the following formula:

σ = P/A(1)

The bulk density of graphene-modified carbon foam composites was measured according to the Archimedes method. The porosity was calculated as follows:

(2) $$C\left(\%\right)=\left(1-\frac{\rho_{v}}{\rho_{s}}\right)\times 100\%$$

where C, $\rho_{v}$ and $\rho_{s}$ are the porosity, the bulk density and the true density of the composites, respectively.

### 2.6. Adsorption Performance Test of Graphene-Modified Carbon Foam Composites

**Figure 1:**
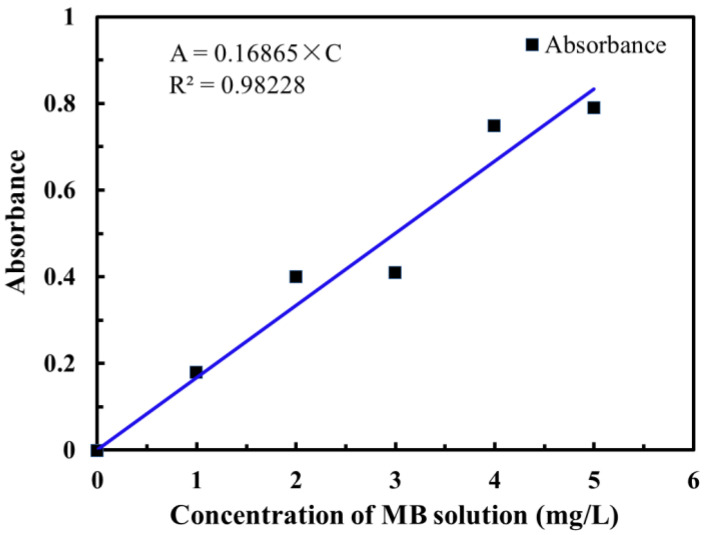
Standard working curve of MB solution.

In this paper, MB solution was taken as the target pollutant to test the adsorption performance of the graphene-modified carbon foam composites. First, the standard concentration curve of the MB solution needed to be calibrated. According to the measurement results of a double-beam ultraviolet–visible photometer, the maximum absorption wavelength of MB is 666 nm. MB solution with concentration 1–5 mg/L and concentration gradient 1 mg/L was prepared, and the corresponding absorbance was measured, respectively. The standard working curve of MB solution with MB concentration as the abscissa and absorbance as the ordinate was drawn, as shown in Figure 1. The equation was obtained by fitting with the linear correlation coefficient R^2^ = 0.98228.

Then, we measured the absorbance of MB with graphene-modified carbon foam composites as adsorbent and calculated the concentration of MB according to the standard working curve of the MB solution; the change of dye concentration was obtained and then the adsorption capacity at time *t* (*q_t_*), the final adsorption capacity (*q_e_*) and the removal rate (*R*) of composite were calculated as follows:

(3) $$q_{t}=\frac{(\rho_{0}-\rho_{t})\times V}{m}$$

(4) $$q_{e}=\frac{(\rho_{0}-\rho_{e})\times V}{m}$$

(5) $$R=\frac{\left(\rho_{0}-\rho_{e}\right)}{\rho_{0}}\times 100\%$$

where $\rho_{0}$ is the initial concentration of MB solution before adsorption (mg/L), $\rho_{t}$ is the concentration of MB solution at time t (mg/L), $\rho_{e}$ is the concentration of MB solution in adsorption equilibrium (mg/L), *V* is the volume of MB solution (L) and *m* is the mass of the adsorbent (mg).

## 3. Results

### 3.1. Microstructure Analysis

**Figure 2:**
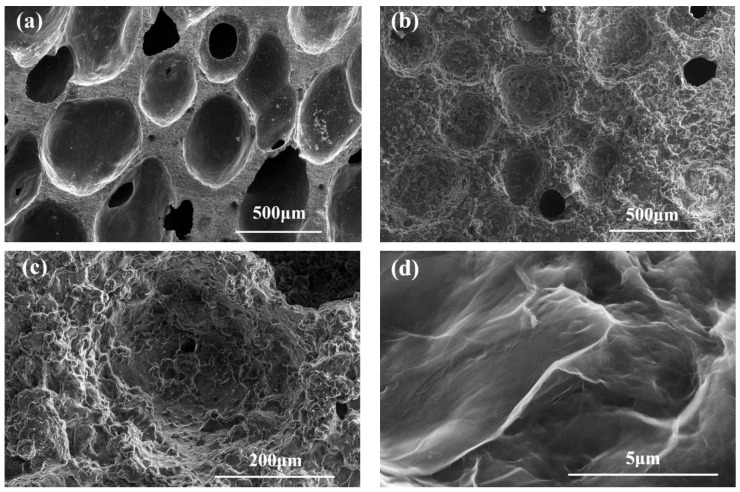
SEM images of (**a**) raw carbon foam, (**b**) graphene-modified carbon foam composites, (**c**) magnification of the single hole, (**d**) focus on the surface of graphene gel.

The SEM images of graphene-modified carbon foam composites are shown in Figure 2. The SEM image of low magnification in Figure 2a clearly reveals that the surface of the raw carbon foam was relatively smooth, with a pore size range of 200–600 μm. There were a few through-holes on the surface. After compounding with graphene gel, as can be seen from Figure 2b, a thick layer of graphene gel was attached to the surface of the carbon foam, and there were few through-holes visible on the top of the composite. Figure 2c is an SEM image obtained by enlarging the pores of the composite to a higher magnification, which indicates that the graphene gel was fully attached to the inner wall of the composite pores, which proves that the attachment method adopted in this study is effective and feasible and results in the good attachment of graphene gel to the cell wall of carbon foam. Figure 2d shows the SEM image obtained by further enlarging the graphene gel on the surface of the composite. It can be seen clearly from the figure that the graphene gel was still in a wrinkled shape, and the wrinkled morphology of the graphene gel further increased the specific surface area of the composite on the basis of porous carbon foam, thus, providing a significant, synergistic effect for improving the adsorption performance of the graphene-modified carbon foam composites.

### 3.2. Crystallographic Analysis 

**Figure 3:**
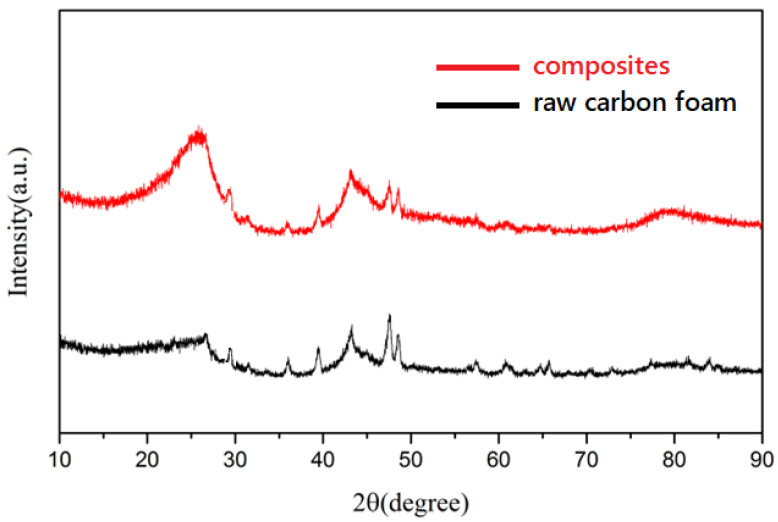
XRD patterns of raw carbon foam and graphene-modified carbon foam composites.

Crystallographic analysis was carried out on the raw carbon foam and graphene-modified carbon foam composites, respectively, and the results are shown in Figure 3. Compared with the XRD pattern of the raw carbon foam, the characteristic peak at 26.7° was more obvious in the XRD pattern of the composite. The peak at this position corresponded to the (002) crystal plane of graphene, which indicates that the graphene gel was successfully attached to the surface of the carbon foam. It can be seen from the figure that the characteristic peak of graphene gel was relatively wide, which indicates that the graphene oxide prepared during the experiment had not completely disappeared, so the spacing between graphene layers was greater than 0.34 nm. On the other hand, after attaching the graphene gel, the characteristic peak intensity of carbon foam in the composite increased, which indicates that the existence of graphene gel improved the disorder of the carbon foam structure.

### 3.3. Raman Analysis

**Figure 4:**
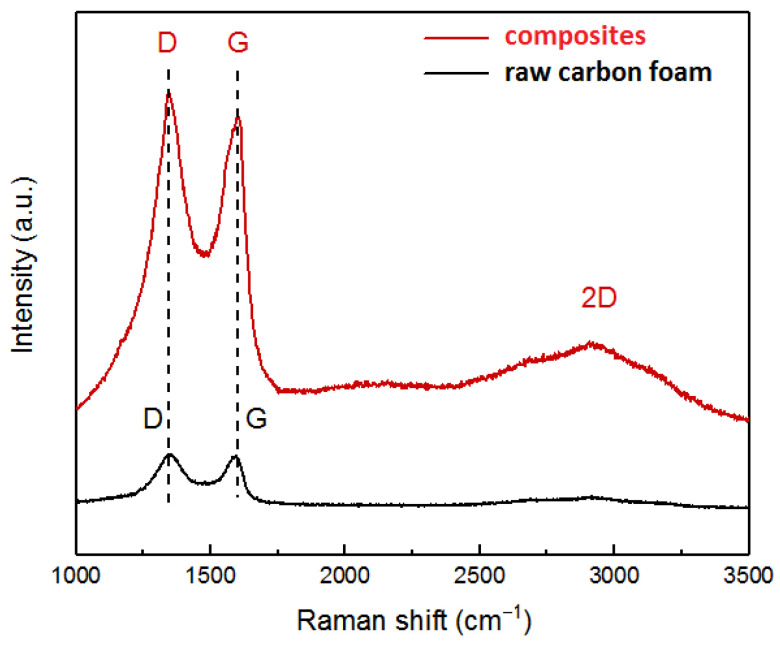
Raman spectrum of raw carbon foam and graphene-modified carbon foam composites.

The Raman spectrum of raw carbon foam and graphene-modified carbon foam composites is shown in Figure 4. Generally, the Raman spectrum of carbon materials contains two main peaks, namely, the D peak and G peak. A 2D peak also exists, which is the secondary response of the D peak. The D peak represents the structural defect of the atoms, and its strength represents the degree of chaos of the carbon atoms. The G peak reflects its symmetry and order, and its intensity represents the content of carbon atoms in the sp^2^ hybrid plane. The 2D peak also represents the degree of atomic confusion. The stacking degree of graphene can be determined by the height of the 2D peak. In addition, the strength ratio of the D peak and G peak, R = I_D_/I_G_, represents the defect degree and graphitization degree of the material. The larger the R value, the more defects of the material and the worse the structural order.

It can be seen from Figure 4 that both the raw carbon foam and the graphene-modified carbon foam composite had peaks near 1300 cm^−1^, 1570 cm^−1^ and 2700 cm^−1^. The D and G peaks of the composite were significantly higher than those of raw carbon foam, while the 2D peak of composites showed a broad peak shape, indicating that the graphene prepared was multilayer. In addition, the R value of the raw carbon foam was 0.98, while the R value of the composite reached 1.15, indicating that the prepared graphene-modified carbon foam composite had more defects, because the graphene had not been fully reduced, and the carbon foam had not been graphitized. These large numbers of defects provided more adsorption active sites, which were conducive to improving the adsorption performance of the composites. 

### 3.4. Analysis of Mechanical Properties

**Figure 5:**
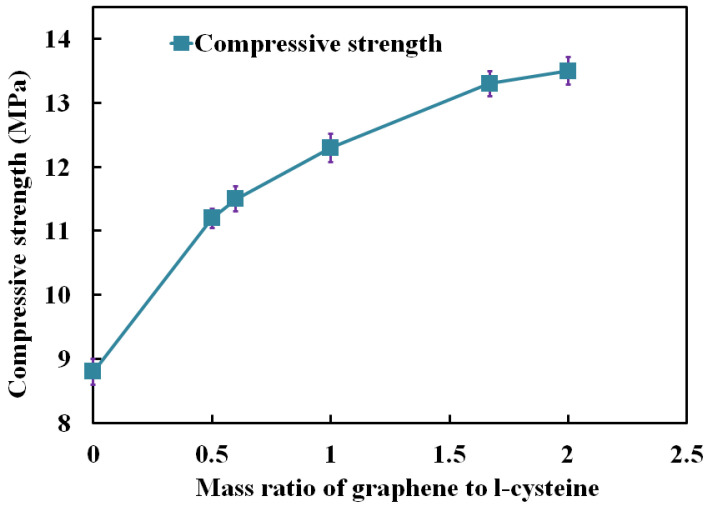
Compressive properties of graphene-modified carbon foam composites.

Figure 5 shows the relationship between the compressive strength of the composite and the mass ratio of graphene to L-cysteine. Notably, the value with the mass ratio of 0 corresponds to raw carbon foam, where compressive strength was 8.8 MPa. With the increasing of graphene content, the compression performance of the composite increased gradually. When the mass ratio of graphene to L-cysteine increased from 0.5 to 2.0, the compressive properties of the composite increased from 11.2 MPa to 13.5 MPa. Compared with raw carbon foam, the maximum compressive strength increased by 53.41%. Therefore, it can be concluded that the addition of graphene improved the compression performance of the composite. On the one hand, when the gel state of graphene was filled into the pores of carbon foam, it enhanced the strength and connectivity of the ligament of carbon foam, thus, enhancing the compression performance. On the other hand, graphene gel had a pinning effect on the cracks of carbon foam, so it alleviated crack propagation, which reduced the stress concentration effect of the cracks and, thus, enhanced the compression performance of the composites. Moreover, based on the good flexibility of graphene sheets, when subjected to external stress, the graphene sheets bent to adapt to the external force. Therefore, the unique properties of graphene can significantly improve the compression properties of composites.

### 3.5. Analysis of Density and Porosity

Table 1 shows the bulk density ($\rho_{v}$), true density ($\rho_{s}$) and porosity (C) of the carbon foam and the composites. The porosity of the raw carbon foam was 46.83%, and the porosity of the composite after adding graphene gel was 58.14%, which was 24.15% higher than that of raw carbon foam. This is because the addition of graphene gel introduced more secondary pores and generated more adsorption sites through its own wrinkles and defects, which eventually increased the porosity and improved the adsorption performance of the composites.

### 3.6. Methylene Blue Adsorption Performance of Graphene-Modified Carbon Foam Composites

#### 3.6.1. Effect of the Graphene-to-L-Cysteine Ratio on Adsorption Performance

**Figure 6:**
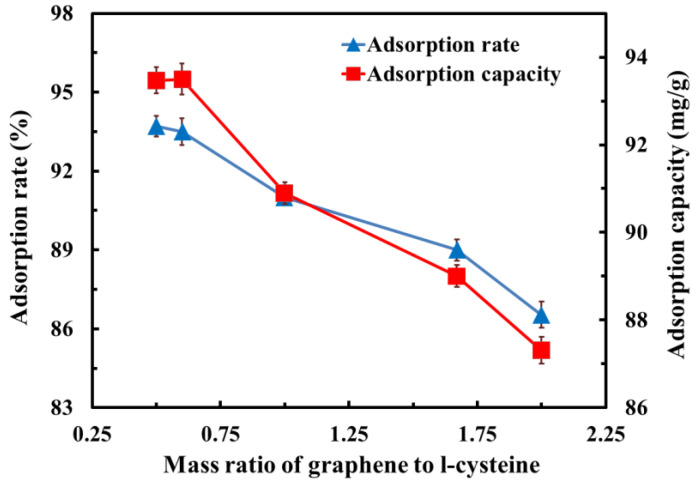
Effect of the graphene-to-L-cysteine ratio on adsorption rate and adsorption capacity.

Figure 6 shows the effect of the graphene-to-L-cysteine ratio on the adsorption rate and capacity of composites. It can be seen from the figure that the adsorption rate and adsorption capacity of the graphene/carbon foam composite showed the same trend as the increasing graphene-to-L-cysteine ratio; the variety of the adsorption rate and adsorption capacity can be divided into two stages with the change of graphene-to-L-cysteine ratio. In the first stage, when the ratio was 0.5–0.6, the adsorption rate and adsorption capacity basically remained the same. In the second stage, the adsorption rate decreased from 93.71% to 86.53%, and the adsorption capacity decreased from 93.47 mg/g to 87.31 mg/g as the ratio gradually increased.

It should be emphasized that the adsorption rate and adsorption capacity of raw carbon foam were 37.33% and 74.65 mg/g, respectively, so the addition of graphene gel effectively improved the adsorption performance of the composites. Compared with raw carbon foam, when the graphene-to-L-cysteine ratio was 0.5, the maximum adsorption rate and adsorption capacity of the modified composites reached 93.71% and 93.47 mg/g, increased by 151% and 25%, respectively. This is because, at the stage when the ratio was 0.5–0.6, the specific surface area of carbon foam was significantly increased by adding graphene gel, and there were many surface active sites of graphene gel which provided more adsorption sites for MB molecules, so the adsorption rate and adsorption capacity were significantly improved. When the ratio reached 0.6, the graphene gel content reached saturation. In the second stage, with the increase in the graphene content, the graphene folds covered each other due to the stacking of multiple layers of graphene. Moreover, too much graphene gel covered the through-hole of carbon foam, which was not conducive to the increase in the specific surface area of the composites, thus, gradually reducing the adsorption rate and adsorption capacity. 

#### 3.6.2. Effect of Adsorbent Addition on Adsorption Performance

**Figure 7:**
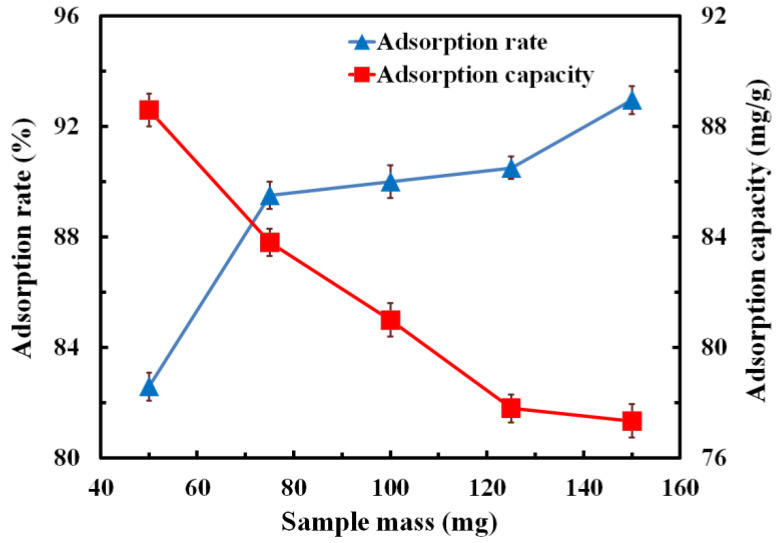
Effect of sample mass on the adsorption rate and adsorption capacity.

The samples of graphene-modified carbon foam composites with masses of 50 mg, 75 mg, 100 mg, 125 mg and 150 mg were used for the adsorption test on the MB solution with concentration of 5 mg/L; the adsorption rate and adsorption capacity of the samples were calculated subsequently. The testing results are shown in Figure 7. It can be seen from the figure that, with the increase in sample mass, the adsorption rate showed a gradual upward trend, from 82.59% to 92.95%, increasing by 12.54%. The adsorption capacity decreased with the increase in sample mass, from 88.59 mg/g to 77.35 mg/g, decreasing by 12.68%. Therefore, as the sample mass increased, the specific surface area of the sample increased gradually, as well as the area of graphene on the composites’ surface, so the number of active sites on the composite surface increased significantly, which caused the increase in the adsorption rate. As for the adsorption capacity, although the adsorption ability increased with the increasing sample mass, the increase in MB adsorption ability of the sample was smaller than the increase in sample mass, which led to the decrease in the adsorption capacity with the increasing sample mass.

#### 3.6.3. Effect of MB Concentration on Adsorption Performance

**Figure 8:**
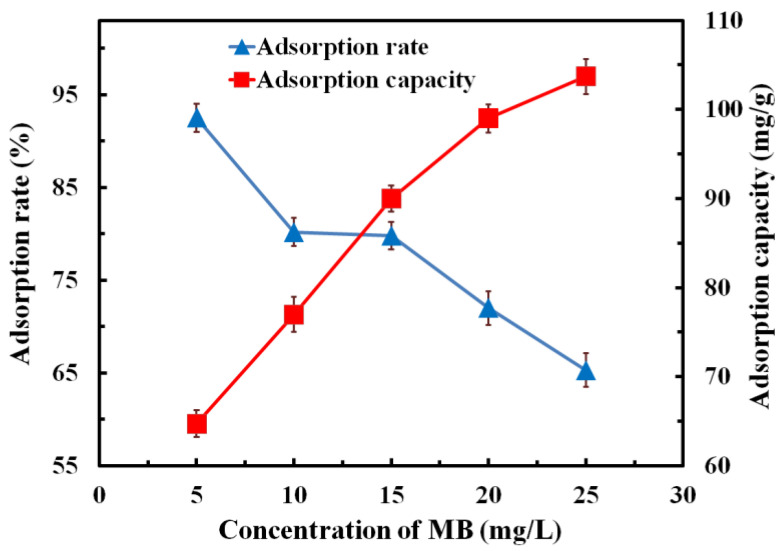
Effect of MB concentration on the adsorption rate and adsorption capacity.

We immersed 150 mg of composites in MB solution with different initial concentrations (5 mg/L, 10 mg/L, 15 mg/L, 20 mg/L, 25 mg/L); after the adsorption equilibrium state was achieved, the relationship between MB solution concentration and adsorption rate/adsorption capacity was calculated, as shown in Figure 8. It can be seen from the figure that the adsorption rate of the sample showed a decreasing trend, while the adsorption capacity showed an increasing trend. With the MB concentration increasing from 5 mg/L to 25 mg/L, the adsorption rate of the composite decreased from 92.51% to 65.32%, and the adsorption capacity increased from 64.73 mg/g to 103.69 mg/g.

It can be inferred that the higher the concentration of MB solution, the more MB molecules. Hence, more MB molecules can contact the sample surface and be captured. Finally, on the premise of the same sample mass, the higher the concentration of MB solution, the more MB molecules are adsorbed on the sample surface, so the adsorption capacity increases. On the other hand, although the number of adsorbed MB molecular increases, the MB solution concentration also increases in a larger amount, so the adsorption rate of the composite to MB solution is reduced.

#### 3.6.4. Effect of pH Value on Adsorption Performance

**Figure 9:**
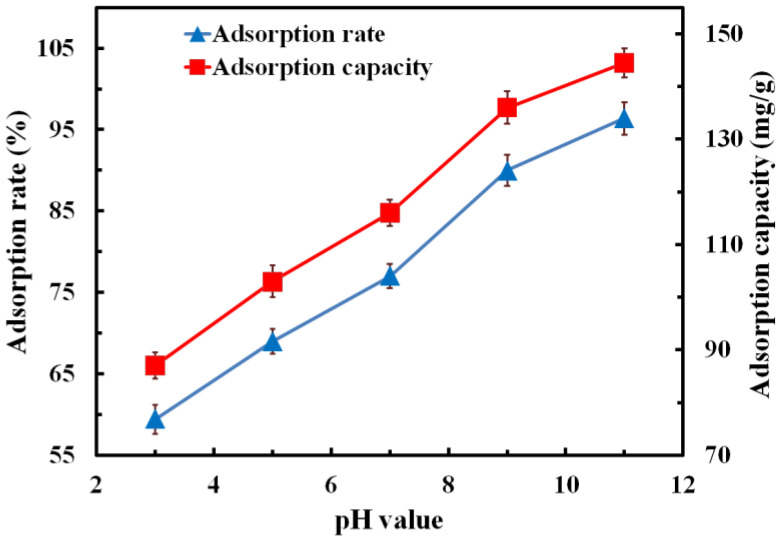
Effect of pH value on the adsorption rate and adsorption capacity.

We immersed 150 mg of composites in MB solution with concentration of 5 mg/L and pH values of 3, 5, 7, 9 and 11, respectively. The relationship between the pH value of the MB solution and the adsorption rate/adsorption capacity is shown in Figure 9. It can be seen from the figure that, with the increase in the pH value, both the adsorption capacity and the adsorption rate showed a trend of gradual increase. When the pH value rose from 3 to 11, the adsorption rate increased from 59.41% to 96.38%, and the adsorption capacity increased from 87.09 mg/g to 144.45 mg/g, increasing by 62.28% and 65.86%, respectively. Therefore, the increase in pH value had a great influence on the adsorption rate and capacity of the composite for MB solution. When the solution was acidic, a large amount of H^+^ in the solution was ionized and attached to the surface of the composites, which made the sample have positive electricity. Because MB is a cationic dye, there was a competitive adsorption phenomenon between ionized H^+^ and MB molecules in the solution. Therefore, the adsorption of the MB molecules on the composites was reduced, and the stronger the acidity, the lower the adsorption of the MB molecules. On the contrary, when the solution was alkaline, a large number of OH^−^ ions in the solution were ionized, which attached to the sample surface to make the composites have negative electricity. OH^−^ ions on the sample surface adsorbed cationic dyes to the surface of the sample through electrostatic action. With the increase in pH value in the MB solution, more OH^−^ ions were ionized from the solution; thus, more OH^−^ ions attached to the surface of the composites. As a result, more MB molecules were adsorbed by the composites, which led to the improvement of the adsorption performance. Compared with raw carbon foam, when the pH value of the MB solution was 11, the maximum adsorption rate and adsorption capacity of the modified composites increased by 158.18% and 93.50%, respectively.

## 4. Conclusions

In this paper, graphene-gel-modified, pitch-based carbon foam composites were synthesized. The microstructure and morphology of the composites were studied by SEM, XRD and Raman spectroscopy; the compressive property and porosity were also tested. The adsorption test was carried out using MB solution to simulate a dye solution. The influence of the composite properties and MB solution on the adsorption performance was investigated. Through the microstructure characterization of the composites, it was found that the surface of the carbon foam was fully covered by graphene, and some graphene oxide was not completely reduced. Raman spectroscopy showed that the graphene gel on the surface of the composite had a multilayer graphene structure with many edge defects, which provided sufficient active adsorption sites and helped to increase the adsorption performance of the composites. With the increase in the mass ratio of graphene to L-cysteine, the compressive properties of the composites also increased. When the mass ratio of graphene to L-cysteine was 2.0, the compressive strength of the composites reached 13.5 MPa, increased by 53.41%. Results showed that the porosity of the composite after graphene modification was 58.14%, which was 24.15% higher than that of raw carbon foam. According to the analysis of the adsorption performance, when the mass of the adsorbent was 150 mg, the initial concentration of the MB solution was 5 mg/L, and the pH value of the MB solution was 11; the composites showed the best adsorption effect. In this case, the adsorption rate was 96.3%, increased by 158.18%, and the adsorption capacity was 144.45 mg/g, increased by 93.50%.

## Figures and Tables

**Table 1:** The density and porosity of the carbon foam and the composites.

Project	$\rho_{v}\;(g/{\mathrm{cm}}^{3})$	$\rho_{s}\;(g/{\mathrm{cm}}^{3})$	C (%)
Raw carbon foam	0.589	1.108	46.83
Composites	0.622	1.486	58.14

## Data Availability

The raw data presented in this study are available on request from the corresponding author.
